# Durable Expression of Minicircle DNA-Liposome-Delivered Androgen Receptor cDNA in Mice with Hepatocellular Carcinoma

**DOI:** 10.1155/2014/156356

**Published:** 2014-03-06

**Authors:** Tian-You Chang, Chin-Ying Chung, Wei-Min Chuang, Long-Yuan Li, Long-Bin Jeng, Wen-Lung Ma

**Affiliations:** ^1^Sex Hormone Research Center, Center for Molecular Medicine, and Organ Transplantation Center, China Medical University Hospital, Taichung 404, Taiwan; ^2^Graduate Institution of Clinical Medical Science, Department of Medicine, School of Medicine, China Medical University, Taichung 404, Taiwan; ^3^Department of Pharmacology, The Pennsylvania State University College of Medicine, Hershey, PA 17033, USA

## Abstract

*Background*. The most common gene-based cancer therapies involve the suppression of oncogenic molecules and enhancement of the expression of tumor-suppressor genes. Studies in noncancer disease animal models have shown that minicircle (MC) DNA vectors are easy to deliver and that the proteins from said MC-carrying DNA vectors are expressed over a long period of time. However, delivery of therapeutic genes via a liposome-mediated, MC DNA complex has never been tested in vascular-rich hepatocellular carcinoma (HCC). Liposome-mediated DNA delivery exhibits high *in vivo* transfection efficiency and minimal systemic immune response, thereby allowing for repetitive interventions. In this study, we evaluated the efficacy of delivering an MC-liposome vector containing a 3.2 kb androgen receptor (AR; HCC metastasis suppressor) cDNA into Hepatitis B Virus- (HBV-) induced HCC mouse livers. *Results*. Protein expression and promoter luciferase assays revealed that liposome-encapsulated MC-AR resulted in abundant functional expression of AR protein (100 kD) for up to two weeks. The AR cDNA was also successfully delivered into normal livers and diseased livers, where it was persistently expressed. In both normal livers and livers with tumors, the expression of AR was detectable for up to 60 days. *Conclusion*. Our results show that an MC/liposome delivery system might improve the efficacy of gene therapy in patients with HCC.

## 1. Introduction

Most solid tumors possess characteristics that are not observed in normal tissues, such as extensive angiogenesis and hence hyper-vasculature, defective vascular architecture, impaired lymphatic drainage/recovery systems, and greatly increased production of a number of permeability mediators [1]. The enhanced permeability and retention (EPR) effect is a phenomenon unique to and ubiquitous in tumors which allows macromolecules such as lipids to enter the tumor interstitial space (enhanced permeability) where they are sequestered due to suppressed lymphatic filtration (retention). The EPR effect provides a great opportunity for more selective targeting of lipid- or polymer-conjugated anticancer drugs, such as SMANCS [2], PK-1 [3], and liposome-encapsulated DNA to the tumor.

The rapid injection of a large volume of DNA solution into a vein was shown by Liu et al. to be an effective method for transfecting DNA into liver, lung, kidney, and heart [4]. The procedure, known as the hydrodynamic method, involves the application of controlled hydrodynamic pressure in capillaries to enhance endothelial and parenchymal cell permeability as a driving force for* in vivo* gene transfer. Gao et al. reported that the injection of a large volume of DNA solution (8~12% of body weight) within a short time (3~5 s) can generate a transfection efficiency of up to 40% in hepatocytes [5]. This method has been used for delivery of DNA that code for small proteins, such as hemophilia factors [6, 7], cytokines [8], hepatic growth factors [9], and alpha1 antitrypsin [10] in mice and rats. Ortaldo et al. showed that hydrodynamic administration of the IL-2 gene resulted in the rapid and transient production of up to 160 ng/mL of IL-2 protein in serum [11]. However, the problem with the hydrodynamic method for gene transfer in humans is that it is not possible to achieve a good transfection efficiency using a DNA solution of up to 12% of body weight within a short period of time [12].

The duration of protein expression from genes delivered via plasmid DNA delivery is normally transient, lasting for only a few days, followed by a period of prolonged yet low level expression. It is thought that unmethylated CpG sequences in the plasmid backbone stimulate intracellular innate immune responses [13, 14]. Plasmids without CpG sequences (i.e., minicircles (MC)) that no longer contain antibiotic resistance markers or the bacterial origin of replication but contain only the functional part of the plasmid have been shown to prolong the duration of protein expression [15]. These small vectors provide for long-term transient expression of multiple transgenes without the risks of immunogenic responses as in standard plasmids. Chen et al. [15] showed that minicircles expressed 45- and 560-fold more serum human factor IX (FIX) and human alpha1-antitrypsin (AAT) than their parent unrecombined plasmids in mouse livers 3 weeks after transgene delivery. Yew et al. [16] showed that transfection of plasmid DNA depleted of nearly 80% of CpG motifs not only resulted in considerably fewer changes in blood parameters, lower levels of inflammatory cytokines, and less liver damage, but also resulted in longer transgene expression in lung and liver of immunocompetent mice than in mice that had been transfected with unmodified vector.

Hundreds of new cationic lipids have been developed since Felgner et al. first reported that a double-chain monovalent quaternary ammonium lipid, N-[17]-N,N,N- trimethylammonium chloride (DOTAP), effectively binds and delivers DNA to cultured cells [18]. The nonimmunogenic nature and ease of industrial production of these cationic lipids make them appealing for gene transfer. Currently, around 13% of gene therapy trials in progress worldwide employ nonviral liposomal vectors for transgene delivery [19]. The LPD (Liposome-Polycation-DNA) developed by Dr. Gao and Huang has shown a promising nucleic acid carrier for* in vivo* transfection [20]. LPD was originally designed to evade the clearance by macrophage in the reticuloendothelial system (RES) and efficiently deliver the cargo nucleic acid to the tumor cells [21–23]. The LPD was reported to cause very little immunotoxicity and negligible cytokine induction in animal tested [21, 22]. Therefore, LPD has the potential to overcome the transfection limitations of hydrodynamic method. We applied the LPD formula for nucleic acid transfection with minor modification in this present study.

Our team has found that androgen receptor (AR), a 100 kD nuclear transcription factor encoded by the AR gene measuring approximately 3.2 kb [24], promotes HCC initiation [25, 26] and suppresses HCC metastasis [27], indicating that AR plays dual roles in HCC progression. In a previous study, we also provided evidence that combining Sorafenib targeted therapy with ectopic AR expression leads to inhibition of HCC metastasis compared to little AR expression [27]. In this study, we used a human-relevant HBV-HCC mouse model developed by Zheng et al. [28] to examine the efficacy of delivering an MC-liposome vector containing a 3.2 kb androgen receptor (AR; HCC metastasis suppressor) cDNA into Hepatitis B Virus- (HBV-) induced HCC mouse livers and tested whether an MC-carrying AR DNA vector results in expression latency in tumors.

## 2. Methods

### 2.1. Construction of AR cDNA into Minicircle Backbone and Mass Production of Minicircle DNA

**Figure 1:**
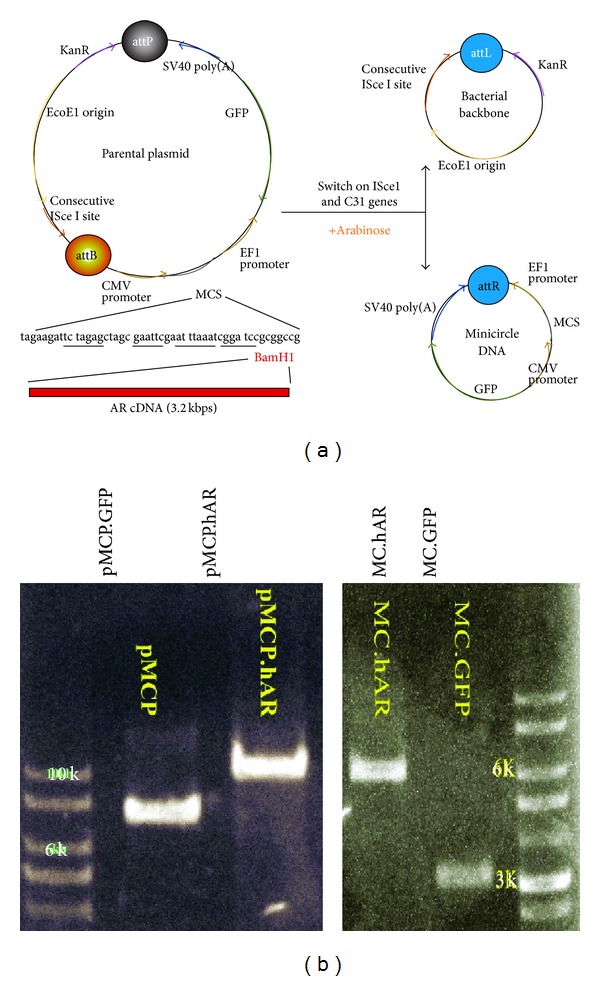
Construction of AR-expressing minicircle vectors and the production of minicircle DNA. (a) Construction of AR-expressing minicircle DNA. Parental plasmid contained antibiotics kanamycin resistance gene and replication EcoE1 Ori cassette flanked by attP and attB site recognized by *φ*C31 intergrase. Upon addition of arabinose, *φ*C31 intergrase and ISce1 endonuclease gene are activated by pBAD operon. The plasmid carries a CMV promoter driven GFP gene and multiple cloning sites (MCS) where AR cDNA is inserted. The 3.2 kbp open-reading frame human AR cDNA was cloned into BamH1 site on MCS to produce a 10 kb AR-expressing plasmid. While producing minicircle DNA, *φ*C31 facilitates the recombination of attP and attB sites and produces two smaller circular DNA fragments, minicircle and bacterial backbone. The backbone plasmid was linearized by ISce1 and further degraded by bacterial endogenous DNase, resulting in a transgene vector (minicircle) without plasmid* ori* and antibiotic resistance genes. (b) GFP minicircle parental plasmids with backbone (pMCP.GFP, 7 kbps; left panel, 2nd lane) and the minicircle DNA (MC.GFP, 3 kbps; right panel, 2nd lane). AR minicircle parental plasmids with backbone (pMCP.hAR, 10 kbps; left panel, 3rd lane) and the minicircle DNA (MC.hAR, 6 kbps; right panel, 1st lane). Reference molecular weight marker for parental plasmids is shown on the left-hand side of the panel. Reference molecular weight marker for minicircle DNAs is shown on the right-hand side of the panel.

Parental plasmid with an expression cassette comprising CMV-MCS-EF1*α*-GFP (SBI) and the kanamycin resistance gene was obtained (System Bioscience, USA). The AR cDNA sequence was released from pBabe.hAR [26] using the restriction enzyme BamH1 and ligated to the parental plasmid using a T4 ligation (NEBL, USA) kit. The resulting plasmid was termed pMCP.hAR and is illustrated in Figure 1(a). In order to produce MC.hAR and MC.GFP DNA, we followed the protocol developed by Kay et al., with slight modifications [29]. In brief, pMCP.hAR and pMCP.GFP were transformed into ZYCY10P3S2T* E. coli *and the cultures were then incubated at 37°C in 5 mL LB (Luria Broth (Lennox); BIO BASIC, CA) containing 50 *μ*g/mL kanamycin for 2 hrs. We then transferred the* E. coli* to a 2-liter flask containing 400 mL fresh TB (Terrific Broth; BIO BASIC, CA) and 50 *μ*g/mL kanamycin and then incubated the culture with shaking (250 rpm) at 37°C for 16~18 hrs. We then added an equal volume of LB containing 2% L-arabinose (Sigma-Aldrich), adjusted the pH to 7, and further incubated the solution at 30°C for 5 hours with shaking. A cesium chloride (CsCl) gradient method was used to precipitate the minicircle DNA.

### 2.2. Preparation of LPD Containing Minicircle DNA

200 *μ*L of LPD for one mouse injection was prepared as previously described [21–23]. Briefly, 24 *μ*g minicircle DNA was condensed by 18 *μ*g protamine through electrostatic interaction into nanometric complexes. The complexes were mixed with 30 *μ*L of 100 nm cationic liposome (20 mM) composed of 1,2-dioleoyl-3-trimethylammonium-propane (DOTAP, Avanti Polar Lipids) and cholesterol (Sigma-Aldrich) at 1 : 1 mol ratio. The LPD nanoparticles were then PEGylated by postinsertion of 30 *μ*L of DSPE-PEG (10 mg/mL) at 50°C for 10 minutes.

### 2.3. Injection of LPD-Containing DNA Complex into HBVtg Mice

A single injection of subminimal dose of hepatic carcinogen diethylnitrosamine (DEN; 2 mg/kg body weight) was given to 12- to 15-day-old HBV-transgenic mice as previously described [28]. The animals were provided by Professor Ou at the University of Southern California [30, 31]. At age 54 weeks, the HBV-lowDEN HCC mice were injected intravenously with 20 *μ*g/100 *μ*L/mouse of LPD (MChAR or MCGFP as control group) twice weekly for 4 consecutive weeks through the caudal vein. The mice were killed two months after minicircle DNA injections. The livers were excised, stored in Tissue-Tek OCT Compound (Sakura; CA90501, USA) for frozen section, and then sliced for GFP signal detection.

### 2.4. *In Vitro* Delivery, Long-Term Culture, Immunoblot Assay, and AR Transactivation Luciferase Assay

In order to evaluate the expression latency of minicircle DNA, MC.GFP or MC.hAR was transfected into 293T cells using a calcium phosphate precipitation method [32]. Once cells grew to confluence, 1 in 10 of the cells was subcultured. GFP expression was detected using a fluorescence microscope (Nikon eclipse 80i) at days 2, 6, 10, and 14. The proteins were harvested 2 days after transfection for detection of AR by western blot [27]. The plasmids pRL-TK (transfection control) and ARE-luciferase (ARE-Luc) reporter were applied in this experiment. Briefly, 1-2 × 10^5^ cells were seeded on 24-well plates 24 hrs before transfection. DHT (10^−8^ M) was added 24 hrs after transfection. Approximately 24 hrs later, cells were harvested and analyzed by the Dual-Luciferase Reporter Assay System (Promega) in combination with a luminometer (Promega).

### 2.5. Histological Examination of Minicircle-Delivered Gene Expression

Sections of normal and tumor livers were embedded in O.C.T. compound (SAKURA, USA) and frozen at −80°C. The tissue sections (8 *μ*m) were then obtained using a microtome (Leica freezing microtome CM1950, Germany). The sections were then fixed with 1.25% glutaraldehyde for 10 min. All sections were inspected and images were acquired using a Nikon Eclipse 80i camera affixed to a fluorescence microscope (Nikon Microscope Eclipse 80i).

## 3. Results

### 3.1. AR Minicircle DNA Construction and Production

In this study, we subcloned a full-length 3.2 kb human AR cDNA into a minicircle parental plasmid (Figure 1(a)). We first linealized pMCP using the BamH1 endonuclease restriction enzyme and then inserted human AR cDNA to generate a 10 kbp pMCP.hAR. To produce minicircle DNA on a larger scale, we performed a two-step incubation of plasmid-transformed ZYCY10P3S2T* E. coli*. The first incubation was done to replicate competent bacteria on a large scale. We then incubated* E. coli* in arabinose-containing medium at a low temperature (30°C) to activate phiC31 and ISce1 genes so that the backbone of the plasmid would be degraded. After incubation, the minicircle DNA was extracted using a CsCl gradient method. The construction and verification of minicircle DNA production are shown in Figure 1(b). The parental plasmids and GFP-carrying minicircle DNA were 7 kbp and 3 kbp (Figure 1(b), left panel). The parental plasmid and AR-carrying miniclrcle DNA were 10 kbp and 7 kbp (Figure 1(b), right panel). Our analyses showed that there was little to no contamination.

**Figure 2:**
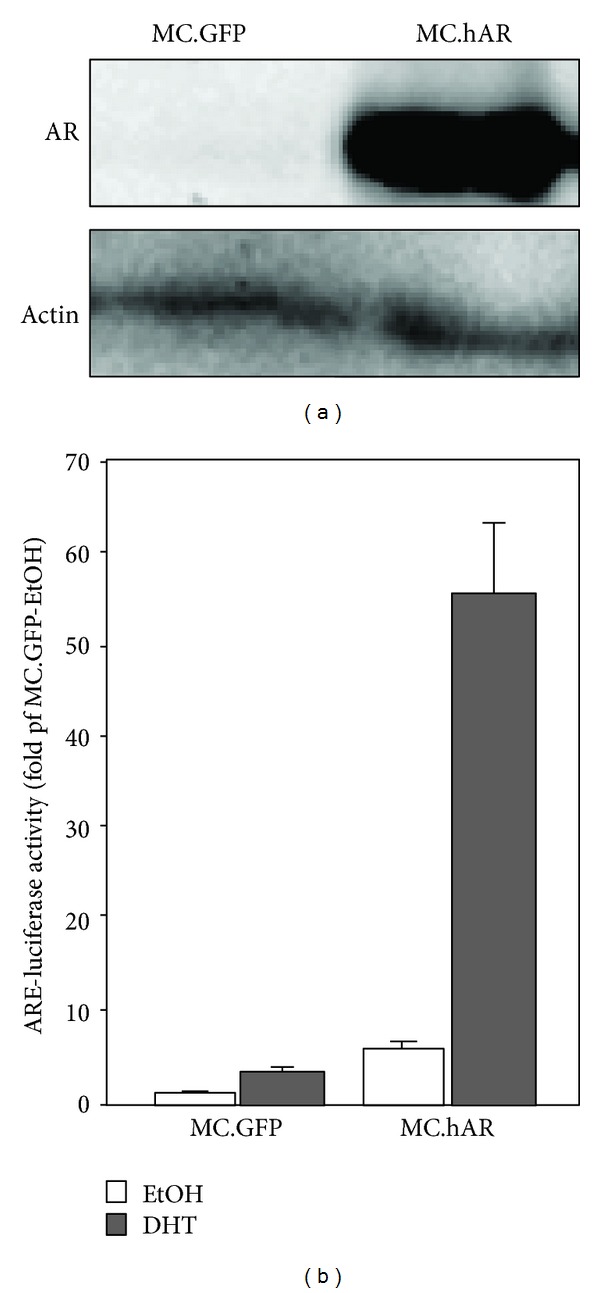
*In vitro* expression and functional analysis of AR minicircle DNA transfection. (a) AR protein expression in the minicircle DNA transfected 293T cells by immunoblot. The left lane is MC.GFP transfectant where the right lane is MC.hAR transfectant. Actin served as the loading control. (b) AR transactivation function was measured by ARE-luciferase assay. The MC.GFP and MC.hAR DNAs were cotransfected with ARE-luciferase into 293T cells and treated with either vehicle (ethanol; EtOH) or dihydrotestosterone (DHT, 10 nM) for 24 hrs. Cell lysate were harvested to analyze the dual-luciferase assay as described in Section 2. The readings of each group were normalized with those of the MC.GFP-EtOH group and plotted on a graph. The data came from at least 3 independent experiments.

In order to verify the expression and activity of AR minicircle DNA, we transiently transfected MC.GFP and MC.hAR into 293T cells. The protein expression of AR was examined by immunoblot assay (Figure 2(a)). We found that AR protein was overexpressed in 293T cells. We then examined AR transactivation to verify the proper folding and function of AR (Figure 2(b)). Treatment of MC.hAR transfectants with 10 nM DHT resulted in robust induction of ARE-luciferase activity compared with ARE-luciferase activity in MC.GFP transfectants.

Taken together, we have demonstrated that our protocol results in the successful construction and production of AR minicircle DNA as well as functional expression of AR protein (Figures 1 and 2).

### 3.2. Durable Expression of Minicircle DNA* In Vitro* and* In Vivo*

**Figure 3:**
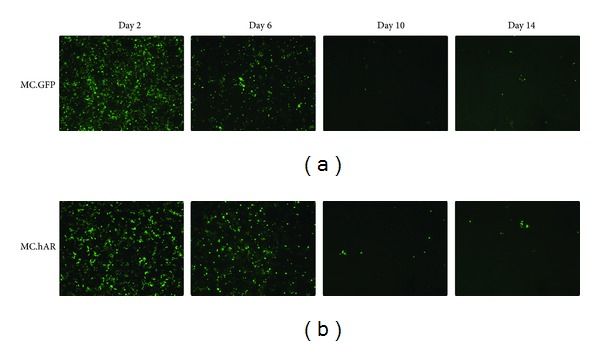
Long-term* in vitro* expression of minicircle DNA in 293T cells. MC.hAR or MC.GFP was transfected into 80% confluence 293T cells, and green fluorescence was observed under fluorescence microscopy at days 2, 6, 10, and 14. The upper panels show MC.GFP transfectant and the lower panels show MC.hAR transfectant. The cells were 1/10 subcultured every three days upon cell reach to around 80~90% confluence. The strong GFP signals were still detectable on day 14 (fourth passage of subcultures).

After establishing our minicircle DNA expression vector, we tested whether AR protein delivered via minicircle DNA could be expressed for an extended period of time. We transfected MC.GFP and MC.hAR in HEK293T cells to measure expression duration (Figure 3). The initial transfection efficiency was approximately 80% (Figure 3, Left panel). We found that GFP expression was still approximately 10% after 4 passages (around 14 days; Figure 3, right panel) of both plasmids. The data suggest that MC DNA : LPD delivery exhibits excellent capacity for long-term expression.

**Figure 4:**
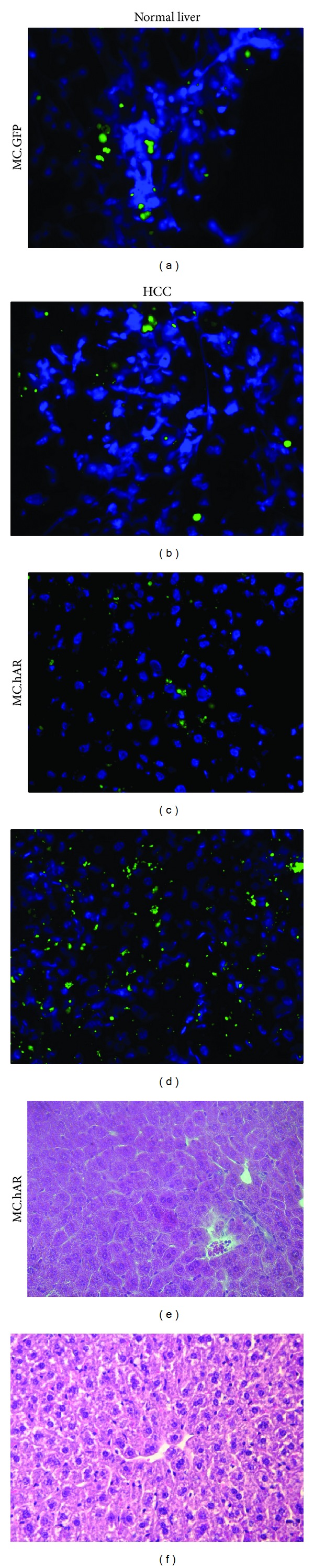
Long-term* in vivo* expression of minicircle DNA in normal and HBV-induced tumor livers. (a) GFP signal of MC.GFP injected normal mouse liver under fluorescence microscope. (b) GFP signals of MC.GFP injected tumor mouse liver under fluorescence microscope. (c) GFP signals of MC.hAR injected normal mouse liver under fluorescence microscope. (d) GFP signals of MC.hAR injected tumor mouse liver under fluorescence microscope. (e) H&E staining image of nontumor counterpart of HBV-HCC mouse after MC.hAR : LPD delivery. (f) H&E staining image of tumor lesion of HBV-HCC mouse after MC.hAR : LPD delivery.

The ideal gene delivery system is one that can efficiently and durably express genes in target organs without chromosome insertion. We, therefore, tested whether AR-carrying minicircle DNA could be expressed in normal livers and liver tumors. We found that minicircle DNA : LPD complex could deliver DNA into normal liver (Figures 4(a), 4(c), and 4(e)) and tumor liver (Figures 4(b), 4(d), and 4(f)). In addition, the GFP signal was detected in livers two months after injection.

Taken together, minicircle DNA exerts durable expression after* in vitro* transfection as well as* in vivo* delivery using Minicircle DNA : LPD complex into normal and tumor liver for 2 months.

## 4. Discussion

### 4.1. Failure of Viral Delivery Systems

Viral-backbone modified vectors can be used as delivery systems for DNA molecules, especially plasmids [33, 34]. For therapeutic purposes, the transgene of interest is assembled in the viral genome, where the virus uses its innate mechanism of infection to enter the cells and release the expression cassette. The gene then enters the nucleus and sometimes integrates into the host genome (i.e., lentiviral vector) and is eventually expressed. Gene expression using viral vectors has been achieved with high transfection efficiencies in organs such as kidney [35], muscle [36], ovary [37], and liver [38, 39]. However, there are several concerns regarding the use of viruses as delivery vectors. The chief concern is the toxicity of the viruses and the potential for generating a strong immune response owing to their capsids. Such toxicities have been observed in numerous animal models [40, 41]. For example, traces of adenovirus titers were detected in seminal fluids of a male patient who had earlier received adenoviral based gene therapy, further compounding the fear of possible germline tampering [42]. Adenoviral vectors used for gene therapy for cystic fibrosis were shown to cause a strong immunogenic response [43]. The death of a patient with respiratory and multiple organ failure participating in a FDA-approved gene therapy clinical trial in 1999 was attributed to lethal immune response to the adenovirus vector used to deliver the gene. This case led to temporary suspension of all gene therapy trials in the United States [44]. Although clinical trials have resumed, this event as well as a few others (Clinical trial ID number: NCT00844623) and (http://clinicaltrials.gov/ct2/results?term=adenoviral±vector%2C±liver&Search=Search) has raised tremendous concern over the safety of using viruses for gene therapy.

In addition, the integration of therapeutic genes into the host genome by a virus takes place in a random fashion. There is no control over the exact location of the insertion of a gene. Random gene insertion can cause insertion mutagenesis that may inhibit expression of normal cellular genes or activate oncogenes, with deleterious consequences [45]. Both these two concerns are not occur while using minicircle DNA : LPD technology for nonviral mediated gene transduction nature, and not chromosome-insertion-sequence contain in minicircle DNA.

### 4.2. Advantages of the Minicircle-LPD Delivery System and Its Potential for Future Cancer Therapy

In contrast to viral delivery systems, lipid nanoparticles are generally less immunogenic owing to the surface PEGylation [46]. However, toxicity related to liposomal gene transfer has been observed. Acute inflammation reactions have been observed in animals treated with airway instillation and intravenous injection of lipoplex [47]. Symptoms include induction of inflammatory cytokines, neutrophil infiltration in lungs, decrease in white cell counts, and in some cases tissue injury in liver and spleen [48]. Part of the inflammatory response seen in treated lungs is related to the unmethylated CpG sequences found in plasmids of bacterial origin. A potent immune stimulant, unmethylated CpG sequences, triggers release of proinflammatory cytokines [14]. Cationic lipids in lipoplexes are capable of enhancing the unmethylated CpG effect [49]. Minicircle DNA, which is devoid of bacterial unmethylated CpG sequences, reduces immune response caused by unmethylated CpG, enhances expression level, and prolongs expression duration [16]. Further, minicircle DNA was condensed by protamine, was wrapped as a core by lipids, and therefore reduced the opportunity of exposing to immune cells to cause immune response. Another advantage of using the LPD delivery system is that LPD tends to accumulate in tumors because of the EPR effect, thus greatly enhancing selectivity [1].

## 5. Conclusion

To the best of our knowledge, this is the first study to use minicircle DNA-LPD to deliver a metastasis suppressor gene in normal and spontaneous-tumor livers. In this study, we used LPD containing minicircle DNA to deliver a 100 kD protein to normal and tumor livers in mice. Our results show that AR protein was robustly expressed for at least two months. The applications of this success would be in the following aspects:

1. cancer therapies usually require a long lasting concentration or expression in the target organ in order to have therapeutic effects. Our model would serve this purpose;
2. one disadvantage of viral-mediated gene therapy would be the systemic immune response caused by viral particle. The LPD carrying minicircle DNA has minimal immune problem which allows for multiple interventions.

## Abbreviations

EPR: Enhanced permeability and retention
HCC: Hepatocellular carcinoma
kbps: Kilo-base pairs
HBV: Hepatitis B Virus
DOTAP: N-[1-(2,3-Dioleoyloxy)propyl]-N,N,N-trimethyl-ammoniummethyl-sulfate
AR: Androgen receptor
GFP: Green fluorescence protein
cDNA: Complementary DNA
kD: Kilodalton
MC: Minicircles
FIX: Factor IX
AAT: Alpha1-antitrypsin
DEN: Diethylnitrosamine
ARE: Androgen response element.
